# Health outcomes in young adulthood among former child refugees in Denmark, Norway and Sweden: A cross-country comparative study

**DOI:** 10.1177/14034948211031408

**Published:** 2021-07-24

**Authors:** Andrea Dunlavy, Karl Gauffin, Lisa Berg, Christopher Jamil De Montgomery, Ryan Europa, Ketil Eide, Henry Ascher, Anders Hjern

**Affiliations:** 1 Centre for Health Equity Studies (CHESS), Department of Public Health Sciences, Stockholm University, Sweden; 2 Danish Research Centre for Migration, Ethnicity and Health (MESU), Department of Public Health, University of Copenhagen, Denmark; 3 Faculty of Health and Social Sciences, Department of Health, Social and Welfare Studies, University of South-Eastern Norway, Norway; 4 School of Public Health and Community Medicine, Institute of Medicine, Sahlgrenska Academy, University of Gothenburg, Sweden; 5 Research Department, Angered Hospital, Sweden; 6 Department of Medicine, Clinical Epidemiology, Karolinska Institutet, Sweden

**Keywords:** Refugee youth, health inequalities, integration policy, mental health

## Abstract

**Aims:**

This study aimed at comparing several health outcomes in young adulthood among child refugees who settled in the different immigration and integration policy contexts of Denmark, Norway and Sweden.

**Methods:**

The study population included refugees born between 1972 and 1997 who immigrated before the age of 18 and settled in the three Nordic countries during 1986–2005. This population was followed up in national registers during 2006–2015 at ages 18–43 years and was compared with native-born majority populations in the same birth cohorts using sex-stratified and age-adjusted regression analyses.

**Results:**

Refugee men in Denmark stood out with a consistent pattern of higher risks for mortality, disability/illness pension, psychiatric care and substance misuse relative to native-born majority Danish men, with risk estimates being higher than comparable estimates observed among refugee men in Norway and Sweden. Refugee men in Sweden and Norway also demonstrated increased risks relative to native-born majority population men for inpatient psychiatric care, and in Sweden also for disability/illness pension. With the exception of increased risk for psychotic disorders, outcomes among refugee women were largely similar to or better than those of native-born majority women in all countries.

**Conclusions:**

**The observed cross-country differences in health indicators among refugees, and the poorer health outcomes of refugee men in Denmark in particular, may be understood in terms of marked differences in Nordic integration policies. However, female refugees in all three countries had better relative health outcomes than refugee men did, suggesting possible sex differentials that warrant further investigation.**

## Introduction

Throughout the past several decades, the Nordic countries have experienced demographic changes to their populations due to international migration. Since the 1970s, refugee and family reunification migration have been among the most common reasons for migration to the Nordic region. Children comprise a substantial proportion of this refugee population, with recent figures showing that up to one third of all refugees seeking asylum in the Nordic countries are younger than 18 [1].

The health and well-being of immigrants is influenced by a confluence of risk and protective factors related to the pre-migration context, the migration experience and post-migration living conditions. Yet, given the nature of forced migration, refugees may experience difficulties that differ from those of other immigrants, including exposure to traumatic events in the country of origin, such as war, violence and perilous migration journeys [2,3], which can continue to influence health and post-migration quality of life in the country of settlement. Refugees also often face considerable post-migration stressors upon arrival, including periods of uncertainty related to asylum status and housing, social relations and access to health care, education and employment. Other post-migration stressors, including labour market marginalisation, discrimination and racism and downward social mobility, can negatively influence refugees’ social position and access to material resources, as well as their health [4].

Refugee children have similarly been shown to have increased risks for poor mental-health outcomes associated with exposure to violence [5] and post-migration stress [6], particularly among those who are newly arrived [6]. Yet, the ways in which adverse health outcomes among refugee children may be exacerbated or alleviated by post-migration factors remains somewhat understudied. Previous longitudinal studies of refugee children in the Nordic region have shown that although high rates of internalising symptoms observed upon arrival decrease over time, the mental-health burden of refugee children remains higher than that found among native-born majority population children [6]. Additional studies have shown patterns of under-utilisation of psychiatric care services among young refugees [7,8], suggesting the presence of barriers to care. Young refugees in the Nordic region also experience greater socio-economic marginalisation and poorer educational attainment outcomes relative to non-refugee immigrant youth and their native-born majority population counterparts [9], which can affect their socio-economic trajectories as well as their health later in life.

**Figure 1.**
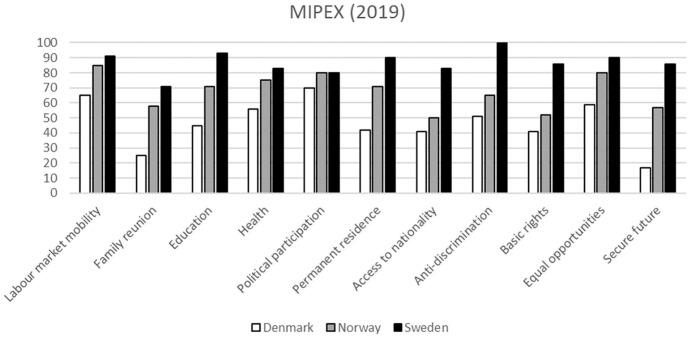
MIPEX ratings of immigration and integration policies in Denmark, Norway and Sweden in 2019.

The extent to which refugees who migrated as children can overcome traumatic pre- and peri-migration experiences as well as post-migration challenges is strongly impacted by the social context in the country of settlement and the resources available to them [10]. The welfare policies of the Nordic countries have many similarities and provide access to health-care services, education and economic benefits for all documented residents. However, in recent decades, there have been substantial and increasing differences between countries within the domain of immigration and integration policy. According to the Migrant Integration Policy Index (MIPEX) [11], a policy analysis tool that evaluates and ranks countries’ immigration and integration policies within multiple policy domains, Denmark stands out from the other Nordic countries, with more restrictive integration policies across all studied policy domains (see Figure 1), and has been classified by MIPEX as having a temporary integration approach [11]. By contrast, Norway and Sweden have been classified as having comprehensive integration policies, with Sweden currently being ranked the highest [11]. Figure 1 shows the MIPEX scores from 2019. Yet, differences between the three countries have been consistent since the first comparative policy ratings were made in 2007 [12].

Unlike pre- and peri-migration factors, those related to the post-migration context are modifiable determinants of immigrants’ health. Cross-country comparative studies of child refugees are particularly relevant for disentangling the role of the post-migration context for several reasons. First, young immigrants arriving to the country of settlement early in life have been exposed to the post-migration policy context for a prolonged period of time during their formative years. As such, comprehensive policy environments that facilitate integration can serve as a buffer against poor health outcomes, while more restrictive environments may create institutional barriers and exclusion that can contribute to the formation of poor health outcomes [13]. Second, immigrant children are less subject to the health-selection effects that have been observed in adult immigrants, who have been shown to have a health advantage relative to native-born populations [14]. Third, previous European studies have shown that differences in health between groups of immigrant children tend to be larger than differences between immigrant children as a whole and their native-born majority population counterparts [15]. As such, comparative examination of the post-migration contexts of refugee children specifically may be particularly important for understanding differences in educational, employment and health outcomes later in life.

The aim of this study was to compare health outcomes of child refugees in young adulthood across three Nordic countries with diverse immigration and integration polices using MIPEX ratings as a platform for discussion of how different immigration and integration policies may directly and indirectly influence health outcomes.

## Methods

### Study populations

Throughout the remainder of the text, the term ‘native-born’ will be used to describe the native-born majority populations of the Nordic countries. The terms ‘refugees’, ‘refugee men’ and ‘refugee women’ will be used to describe individuals who migrated to the Nordic region during childhood as refugees. This includes both accompanied and unaccompanied minors who themselves were granted asylum on refugee grounds, as well as children who arrived as family relations to parents who had been granted asylum on such grounds.

The refugee study population consisted of 27,413 refugees in Denmark, 17,160 in Norway and 109,408 in Sweden who were born between 1972 and 1997 and who were granted residency as children (0–17 years) between 1986 and 2005 (1990–2005 for refugees in Norway). Refugees’ health outcomes were studied from age 18 onwards during the period 2006–2015 and compared to the native-born population birth cohorts in the country of residence.

National population registers in the three countries were used to compile information on demographics, income, education, medical care and cause of death [16]. These data sources were linked on the individual level through a unique personal ID code and then anonymised before data were retrieved by the researchers. The study was approved by the Regional Ethics Committee in Stockholm (Dnr 2013/811-33) in Sweden and the Norwegian Centre for Research Data (project number 47733) and the Norwegian Data Protection Authority (17/00058-3/CDG) in Norway. Processing of the Danish data was authorised by the Office of Research and Innovation at the Faculty of Health and Medical Sciences at the University of Copenhagen (case number SUND-2016-65).

### Health outcomes

Six health outcomes were defined using ICD-10 diagnostic codes and included (a) all-cause mortality (between the ages of 18 and 43); (b) disability/illness pension due to disability or chronic illness at the age of 30 (limited to birth cohorts born between 1972 and 1985); and (c) four indicators of psychiatric care (between the ages of 18 and 43), including any inpatient psychiatric care, any outpatient psychiatric care, any inpatient care with a diagnosis of a psychotic disorder and any inpatient care with a diagnosis related to substance misuse.

### Statistical analysis

The health indicators used in this study depend to a considerable degree on the national context. As such, relative measures that make comparisons with the native-born populations in each country were used for cross-country comparisons. Cox regression was used to analyse risk of mortality and psychiatric care in the refugee populations relative to the native-born comparison populations. These analyses were based on person-time in the study, which was calculated from 1 January 2006 or the 18th birthday (whichever came last) to 31 December 2015, date of death or first outcome (whichever came first). These results are presented as hazard ratios (HR) with 95% confidence intervals (CI). Logistic regression was used to standardise disability/illness pension receipt at the age of 30, with results presented as percentages and odds ratios (OR). All analyses were sex stratified and adjusted for year of birth.

## Results

Table I shows the socio-demographic characteristics of the refugee study populations. The main countries of origin were quite similar across the three Nordic countries, with former Yugoslavia being the most common (see Table I). Most refugee children obtained residency between the ages of 6 and 12 years (41–48%). Refugees in Sweden had the highest proportions of completed secondary education at the age of 25, while the highest proportions of core labour force participation at the age of 25 were observed in Norway. Refugees in Denmark had the highest proportions of being Not in Education, Employment, or Training (NEET) at the age of 25.

**Table I.** Socio-demographic characteristics of the refugee study populations.

	Men			Women		
	Denmark	Norway	Sweden	Denmark	Norway	Sweden
	*N*=15,149	*N*=9446	*N*=57,771	*N*=12,264	*N*=7714	*N*=51,637
	%	%	%	%	%	%
Year of birth						
1972–1976	5.2	5.0	7.3	5.4	6.6	5.9
1977–1981	13.7	11.8	17.6	14.2	11.9	17.2
1982–1985	20.1	20.7	22.2	18.5	19.1	22.2
1986–1989	25.2	26.1	25.3	23.8	24.6	25.8
1990–1993	21.5	21.3	18.0	21.3	24.0	18.5
1994–1997	14.3	15.1	9.6	16.7	16.9	10.4
Age when residency granted						
0–5 years	25.2	24.8	23.9	26.3	28.7	24.8
6–12 years	48.2	41.0	47.6	48.4	43.8	48.1
13–17 years	26.6	34.2	28.5	25.3	27.5	27.0
Country of origin						
Afghanistan	12.7	7.1	3.0	12.2	5.2	2.5
Iran	6.4	13.2	10.0	5.8	11.7	9.0
Iraq	20.8	7.6	15.0	20.6	7.8	13.3
Somalia	10.9	8.6	4.9	10.3	7.6	4.5
Former Yugoslavia	22.0	34.9	19.0	23.8	38.4	18.6
Other	27.2	28.7	50.2	27.3	29.2	51.1
Completed upper secondary education at the age of 25^a^	46.6	42.9	74.6	51.1	56.4	79.6
Core labour force participation at the age of 25^a^	20.1	39.2	31.8	10.9	22.1	20.6
Not in education, employment, or training (NEET) at the of age 25^a^	25.0	15.7	21.3	25.9	20.6	21.3

a Limited to birth cohorts 1972–1990.

### Mortality

Table II presents the results for mortality risk among refugees relative to their native-born counterparts across the three Nordic countries. Mortality risks among refugee women in Denmark and Norway were similar to those of the corresponding native-born populations, while refugee women in Sweden had a slightly lower risk (HR=0.75; 95% CI 0.61–0.95). Refugee men in Denmark had a higher mortality risk than their native-born counterparts (HR=1.59; 95% CI 1.23–2.05), while refugee men in Norway and Sweden had mortality risks that were similar to those who were native-born. The higher mortality risk in refugee men in Denmark was particularly pronounced for external causes (ICD-10 diagnostic codes V00–Y99; HR=1.77; 95% CI 1.27–2.48) compared to mortality related to other causes (HR=1.38; 95% CI 0.93–2.06; data not shown in the table).

**Table II.** Mortality during 2006–2015.

	Men			Women		
	Person-years	Mortality rate/10,000 person-years	HR (95% CI)	Person -years	Mortality rate/10,000 person-years	HR (95% CI)
Denmark						
Native-born	5,540,232	3.6	1	5,282,726	2.2	1
Refugees	123,332	4.9	**1.59 (1.23–2.05)**	97,909	1.5	0.92 (0.55–1.54)
Norway						
Native-born	5,249,213	8.4	1	4,942,294	3.6	1
Refugees	93,659	6.8	0.88 (0.67–1.16)	67,558	3.0	0.91 (0.56–1.47)
Sweden						
Native-born	8,923,267	6.8	1	8,382,545	3.1	1
Refugees	498,090	6.3	0.93 (0.83–1.04)	441,586	2.1	**0.75 (0.61–0.95)**

HR: hazard ratio; CI: confidence interval.

### Disability/illness pension

Table III shows the results for disability pension at the age of 30. Refugee men from Denmark had the highest prevalence rate (4.9%) and an OR of 2.31 (95% CI 2.06–2.59) compared with native-born Danish men. The prevalence among refugee women in Denmark was lower than refugee men (3.0%), but risks were higher relative to native-born women (OR=1.42; 95% CI 1.20–1.68). Refugee men in Sweden also had an increased risk compared to native-born men (OR=1.46; 95% CI 1.35–1.57), while risks were similar to the corresponding native-born populations among refugee women and men from Norway and among refugee women from Sweden.

**Table III.** Disability/illness pension receipt at age 30.

	Men			Women		
	*n*	%	OR (95% CI)	*n*	%	OR (95% CI)
Denmark						
Native-born	406,054	2.2	1	386,619	2.0	1
Refugees	7092	4.9	**2.31 (2.06–2.59)**	5532	3.0	**1.42 (1.20–1.68)**
Norway						
Native-born	335,784	2.3	1	316,020	2.2	1
Refugees	3548	2.7	1.11 (0.92–1.33)	2662	2.0	0.91 (0.70–1.14)
Sweden						
Native-born	560,156	2.2	1	523,913	2.7	1
Refugees	27,207	3.0	**1.46 (1.35–1.57)**	23,413	2.4	0.99 (0.91–1.07)

OR: odds ratio; CI: confidence interval. Bold text indicates results that were statistically significant (*p* < 0.05).

### Psychiatric care

Table IV presents outcomes for inpatient and outpatient psychiatric care. Refugee men in Denmark (HR=1.69; 95% CI 1.56–1.83), Norway (HR=1.28; 95% CI 1.14–1.45) and Sweden (HR=1.27; 95% CI 1.31–1.34) had higher risks than their native-born counterparts for having been admitted at least once for inpatient treatment with a main psychiatric disorder, while risks for refugee women in Norway and Denmark were similar to the corresponding native-born populations. In Sweden, refugee women had a lower risk (HR=0.92; 95% CI 0.87–0.96) than native-born women.

**Table IV.** Inpatient and outpatient care with a main psychiatric diagnosis.

	Men		Women	
	Rate/1,000 person-years	HR (95% CI)	Rate/1,000 person-years	HR (95% CI)
*Inpatient care*				
Denmark				
Native-born	3.0	1	3.7	1
Refugees	5.3	**1.69 (1.56–1.83)**	4.3	1.03 (0.93–1.13)
Norway				
Native-born	2.7	1	3.6	1
Refugees	3.6	**1.28 (1.14–1.45)**	4.3	1.09 (0.96–1.23)
Sweden				
Native-born	2.5	1	3.4	1
Refugees	3.3	**1.27 (1.31–1.34)**	3.3	**0.92 (0.87–0.96)**
*Outpatient care*				
Denmark				
Native-born	8.8	1	13.6	1
Refugees	13.3	**1.37 (1.30–1.44)**	15.1	0.97 (0.92–1.02)
Norway				
Native-born	13.5	1	23.6	1
Refugees	15.8	**1.13 (1.07–1.20)**	23.8	0.95 (0.90–1.0)
Sweden				
Native-born	12.0	1	17.5	1
Refugees	12.1	**0.88 (0.86–0.90)**	15.9	0.99 (0.96–1.02)
*Inpatient care for psychotic disorders*				
Denmark				
Native-born	1.0	1	0.8	1
Refugees	3.3	**3.24 (2.92–3.59)**	1.2	**1.36 (1.13–1.64)**
Norway				
Native-born	0.8	1	0.5	1
Refugees	1.9	**2.53 (2.14–2.99)**	0.9	**2.08 (1.60–2.70)**
Sweden				
Native-born	0.6	1	0.4	1
Refugees	1.5	**2.86 (2.65–3.09)**	0.6	**1.84 (1.63–2.08)**
*Inpatient care with substance misuse diagnosis*				
Denmark^a^				
Native-born	0.5	1	0.2	1
Refugees	1.4	**2.29 (1.96–2.67)**	0.2	1.26 (0.82–1.92)
Norway^b^				
Native-born	0.5	1	0.2	1
Refugees	0.6	1.18 (0.88–1.59)	0.1	0.55 (0.26–1.16)
Sweden				
Native-born	1.4	1	0.7	1
Refugees	2.2	**1.48 (1.39–1.58)**	0.6	**0.69 (0.61–0.79)**

a Main diagnosis only.

b Did not include medical consequences of substance abuse.HR: hazard ratio; CI: confidence interval. Bold text indicates results that were statistically significant (*p* < 0.05).

For psychiatric outpatient care, refugee men in Denmark had the highest risk compared to the native-born (HR=1.37; 95% CI 1.30–1.44). Relative to their native-born counterparts, refugee men in Norway also showed elevated risk (HR=1.13; 95% CI 1.07–1.20), while refugee men from Sweden and refugee women from all countries had lower or similar risks, respectively.

Refugee men and women had higher risks for inpatient care for psychotic disorders relative to the native-born in all countries. Elevated risks relative to the native-born were higher among refugee men than they were for refugee women in Denmark and Sweden, but the sex discrepancy was particularly pronounced when comparing refugee men (HR=3.24; 95% CI 2.92–3.59) and women (HR=1.36; 95% CI 1.13–1.64) in Denmark.

Refugee men in Denmark had the highest risk of hospital admissions with a main or complimentary substance misuse diagnosis relative to the native-born (HR=2.29; 95% CI 1.96–2.67), but refugee men in Sweden also showed elevated risk (HR=1.48; 95% CI 1.39–1.58). Refugee men and women from Norway and refugee women from Denmark had risks similar to the native-born populations, while refugee women in Sweden showed decreased risk (HR=0.69; 95% CI 0.61–0.79).

## Discussion

This study of national cohorts of refugees who immigrated to Denmark, Norway and Sweden as children between 1986 and 2005 has described mortality, disability/illness pension and psychiatric care outcomes among refugees in young adulthood relative to their native-born counterparts. In all three countries, refugee men had higher risks of inpatient psychiatric care compared to native-born men, and refugees of both sexes had higher risks for inpatient care for psychotic disorders relative to native-born comparison populations. Refugee women generally demonstrated health outcomes that were more similar to the native-born populations than did refugee men in all countries. Refugee men from Denmark showed the highest risks for mortality, disability/illness pension, outpatient psychiatric care and inpatient care for substance misuse relative to the native-born.

There are three main findings of this study: (a) with some notable exceptions, the risk for adverse health outcomes was generally higher among refugees compared to the native-born, (b) this pattern was largely driven by higher risks among refugee men than refugee women and (c) refugee men in Denmark had considerably higher risks compared to refugee men in Norway and Sweden.

The poorer health outcomes observed among refugee men in Denmark could potentially be interpreted as better access to health-care services in Denmark. However, this seems unlikely, given the overall pattern of poorer health outcomes observed, including increased risk of mortality and disability/illness pension, indicators that are not confounded by barriers to care. In addition, the pattern of poorer health outcomes coincides with poorer educational attainment and labour market outcomes in Denmark, suggesting that social determinants such as these may have a negative influence on refugees’ health and well-being. It is also unlikely that these findings can be attributed to negative health selection. Despite some socio-demographic heterogeneity among the refugee study populations, previous population-based studies have shown comparable proportions of mental-health symptoms among refugee children who migrated to the Nordic region during the same time as those included in our study [17,18], providing evidence that the poorer health outcomes observed among refugees in Denmark are not due to negative health selection. Additional research from Denmark has shown that individual attributes and abilities (i.e. education) rather than the need for asylum (i.e. experiences of war, human rights violations) have predicted decisions to grant asylum [19]. As such, it is plausible that such practices could actually lead to a selection of more socially advantaged, and potentially healthier, refugees in Denmark. Despite this, our findings showed the poorest health outcomes among refugees in Denmark; by extension, this suggests that restrictive policy contexts may have a particularly detrimental health effect, whereby initial advantages diminish and health disadvantages arise over time.

Policy differences were not a subject of empirical investigation in this study. Yet, the results may be considered in terms of the lower Danish MIPEX scores, indicative of an unfavourable policy environment for refugee integration. The policy environment may affect refugees’ health through multiple pathways [20]. First, disadvantaged educational and employment positions may have an adverse effect on health. As demonstrated in Table I, refugees in Denmark more seldom achieved a secondary education by the age of 25 than refugees in Sweden and had a more vulnerable position on the labour market than refugees in Norway and Sweden did. This coincides with Denmark’s lower relative MIPEX scores for labour market mobility and education. Previous research shows a strong link between employment and educational status and psychiatric and substance misuse disorders in youth and young adulthood [21,22]. Capacities within schools and health-care systems to address the psychosocial needs of refugee children may also have important consequences for their retention in education and for whether these vulnerabilities persist into adulthood.

Second, Denmark’s lower MIPEX scores with regard to family reunification, anti-discrimination, access to nationality and a secure future are also important to consider. Experiences of discrimination [23], social exclusion [24,25] and uncertainty about the future [24] are all important drivers of health problems, and also influence quality of life [26]. These factors could represent intersecting pathways through which restrictive immigration policies may exacerbate health vulnerabilities among refugees in Denmark [13,27]. Further studies are needed to test this hypothesis, particularly in light of Denmark’s increasingly restrictive integration policy context that is focused on temporary residence followed by deportation or ‘repatriation’ rather than permanent integration [11]. This may exacerbate stressors related to refugees’ post-migration security and negatively influence health.

Clear sex differentials in the health indicators were also observed. While refugee men in all countries had equal or poorer health outcomes relative to their male native-born counterparts, the differences between refugee women and native-born women were generally less pronounced, and refugee women also tended to show lower risks for poor health than refugee men. The extent to which differences between refugee men and women could be attributed to the policy environment is uncertain. However, the mechanisms through which the post-migration context influences health may be different for refugee men and women. Previous research has pointed to sex differences in achievement–expectation mismatch, social marginalisation and substance use as possible explanations for elevated risk of psychotic disorders among refugee men relative to refugee women [28]. Other studies have similarly suggested that experiences of discrimination are highly gendered, whereby racial and ethnic minority men may be subjected to a greater amount of discrimination than women [29]. This in turn could translate into a relatively larger health disadvantage among refugee men. More studies are needed to elucidate sex disparities better in experiences of post-migration stressors as they relate to health.

### Limitations

Despite our use of total population cohorts, the low frequency of the outcomes assessed limited our ability to adjust for multiple covariates or further stratify the analyses. In addition, with the exception of mortality, we assessed indicators of health care and social benefits usage rather than health outcomes themselves. There was also some degree of heterogeneity by origin among refugees settled in each of the compared Nordic countries, which may limit cross-country comparability. The inpatient mentalhealth indicators used in this study reflected care for severe psychiatric disorders, which tend to be more common in men; it is possible that other types of health indicators (e.g. self-report) would have shown different patterns by sex and country of residence. The observational study design utilised also entails that the causal mechanisms between immigration and integration policy contexts and health can only be speculated upon. Further studies are needed to investigate whether restrictive policy changes have had a detrimental impact on the health of refugees and to address the limitations described above.

## Conclusions

This cross-country comparative study of three Nordic welfare states demonstrates that young adult refugee men in Denmark – the country with the most restrictive immigration policy – have poorer health than native-born Danish men, as well as young refugee men in Sweden and Norway. Recent trends towards more restrictive immigration policies [30] in all three countries warrant further study of the associated health consequences of such changes.
